# Pulmonary vein parameters are similar or better predictors than left atrial diameter for paroxysmal atrial fibrillation after cryoablation

**DOI:** 10.1590/1414-431X20198446

**Published:** 2019-09-02

**Authors:** Bolin Li, Honglan Ma, Huihui Guo, Peng Liu, Yue Wu, Lihong Fan, Yumeng Cao, Zhijie Jian, Chaofeng Sun, Hongbing Li

**Affiliations:** 1 Department of Cardiovascular Medicine, The First Affiliated Hospital of Xi'an Jiaotong University, Xi'an, Shaanxi, China; 2 Department of Cardiovascular Medicine, The First Affiliated Hospital of Xi'an Medical College, Xi'an, Shaanxi, China

**Keywords:** Atrial fibrillation, Cryoablation, Right inferior pulmonary vein, Pulmonary vein parameter

## Abstract

Left atrial diameter (LAD) has been considered an independent risk factor for atrial fibrillation (AF) relapse after pulmonary vein isolation (PVI). However, whether LAD or other factors are more predictive of late recurrence in patients with paroxysmal AF remains unclear. We aimed to evaluate the value of pulmonary vein (PV) parameters for predicting AF relapse 1 year after patients underwent cryoablation for paroxysmal AF. Ninety-seven patients with paroxysmal AF who underwent PVI successfully were included. PV parameters were measured through computed tomography scans prior to PVI. A total of 28 patients had recurrence of AF at one-year follow-up. The impact of several variables on recurrence was evaluated in multivariate analyses. LAD and the time from first diagnosis of AF to ablation maintained its significance in predicting the relapse of AF after relevant adjustments in multivariate analysis. When major diameter of right inferior pulmonary vein (RIPV) (net reclassification improvement (NRI) 0.179, CI=0.031–0.326, P<0.05) and cross-sectional area (CSA) of RIPV (NRI: 0.122, CI=0.004–0.240, P<0.05) entered the AF risk model separately, the added predictive capacity was large. The accuracy of the two parameters in predicting recurrence of AF were not inferior (AUC: 0.665 and 0.659, respectively) to echocardiographic LAD (AUC: 0.663). The inclusion of either RIPV major diameter or CSA of RIPV in the model increased the C-index (0.766 and 0.758, respectively). We concluded that major diameter of RIPV had predictive capacity similar to or even better than that of LAD for predicting AF relapse after cryoablation PVI.

## Introduction

Atrial fibrillation (AF) is a fairly common arrhythmia worldwide, with an estimated 33 million people suffering from this condition (1). Pulmonary vein isolation (PVI), according to guidelines (2), is recommended for symptomatic, drug-refractory, paroxysmal AF. As cryoablation has recently been introduced in patients with paroxysmal AF, this procedure may allow a simpler approach to electrically isolate the pulmonary vein (PV) from the left atrium (LA) to prevent recurrent AF. Short-to-midterm studies employing cryoballoons have shown recurrence rates after ablation ranging from 67 to 83.6% for patients with drug-refractory paroxysmal AF (3–5). The reason for AF relapse is not completely understood but is related to restored PV conduction (6,7). It is accepted that reestablishment of conduction is mainly due to gaps between the ablated lesions or less-than-optimal lesion depth, which has a considerable effect on recurrence (8). The extent to which anatomic parameters of the PV might predict electrical reconnection has been shown in a few studies (9–11).

Most research documented the long-term prognostic value of LA diameter or morphology deformities. In fact, outcomes of ablation are highly dependent on atrial disease stage, and the timing of chamber dilation may happen later than regional deformities (12). However, there is no consensus on the influence of increased PV ostium diameter, size, or roundness on the clinical efficacy of cryothermal PVI techniques. The goal of our study was to assess the value of these PV parameters for predicting the risk of recurrence of paroxysmal AF in patients who underwent cryoablation.

## Material and Methods

For this prospective observational study, we enrolled 100 patients (October 2014 to June 2017) with symptomatic paroxysmal AF who had or had not taken antiarrhythmic drugs owing to refusal of chronic drug therapy. This study of cryoballoon ablation versus radiofrequency ablation for paroxysmal AF was performed in the Cardiology Department of the First Affiliated Hospital of Xi'an JiaoTong University and registered at the Chinese Clinical Trial Registry (ChiCTR1800016314). As per the 2014 American Heart Association/American College of Cardiology/Heart Rhythm Society (AHA/ACC/HRS) AF guidelines (13), paroxysmal AF was defined as AF that terminated spontaneously or with intervention within 7 days of onset and that may recur with variable frequency. Medical histories were obtained during clinic visits, and all medical records including electrocardiograms (ECGs) and Holter ECG recordings showing AF episodes were reviewed. The study protocol (conforming to the ethical guidelines of the Declaration of Helsinki) was approved by the institutional ethics committee. We excluded patients who presented with moderate-to-severe valvular stenosis or regurgitation, acute reversible causes of AF, previous AF ablation procedures, myocardial infarction that occurred within 3 months, severe respiratory insufficiency, left atrial thrombi, left ventricular ejection fraction <40%, anatomic variations of the number of PV ostia, pregnancy, or New York Heart Association class III or IV heart disease.

For each patient, all images were obtained with 256-slice computed tomography (CT) scan (Brilliance-i CT; Philips Healthcare, USA) following intravenous injection of non-ionic contrast medium (Iohexol Injection, 100 mL at 5 mL/s, 128 mm×0.625 mm collimation). Images were obtained from the level of the diaphragm to the aortic arch scanning in high-pitch spiral acquisition mode during a single breath-hold. Prospective electrocardiographic gating was considered to eliminate cardiac motion artifact. Image reconstruction was accomplished by a post-processing workstation using 2D viewing modes and 3D reconstruction. After 3D and 2D images were acquired, the right and left PVs were identified, and the maximum and minimum diameters of the PV ostia were measured in optimal images. The form of the PV ostia was identified by using a venous ostium index, which was calculated by dividing the minor axis by the major axis for each PV. The cross-sectional area (CSA) was calculated as p × (major axis / 2 × (minor axis / 2) for each PV (14).

Cryoablation was performed under local anesthesia by one of two operators. Briefly, a bolus of heparin was administered intravenously preceding transseptal puncture to maintain an activated clotting time between 250 to 350 s. A deflectable transseptal guiding introducer (8 F, 2.6 mm, St. Jude Medical, USA) was inserted via free-hand technique into the right subclavian vein and placed in the coronary sinus to guide the transseptal needle (1.3/0.5 mm, St. Jude Medical) to successfully puncture the atrial septum. Then, a second-generation cryoballoon (Medtronic CryoCath LP, Canada) was introduced into the LA. Once the balloon was expanded in the PV, occlusion was assessed with 50% diluted contrast injection. Subsequently, cryoenergy was delivered to each PV for two freezing cycles of less than 180 s. We began to operate more often in the left superior pulmonary vein (LSPV) than in the left inferior (LIPV), right superior (RSPV), or right inferior pulmonary vein (RIPV). Finally, PV conduction was re-evaluated with the Achieve catheter (Medtronic Inc., USA). Successful PVI was considered isolation of all the PV potentials recorded from the Achieve catheter. For perioperative AF without conversion to sinus rhythm, direct current cardioversion was performed.

Evidence of atrial thrombus formation was evaluated by transesophageal echocardiography. M-mode, 2-dimensional, and Doppler echocardiography were performed and analyzed by 2 cardiologists in accordance with the American Society of Echocardiography guidelines (15) For each patient, laboratory tests were conducted after fasting for 12 h. All patients were informed of the necessity and risks of cryoballoon ablation, and they all provided written informed consent before the ablation procedure.

After successful PVI, the patients were sent back to the ward and monitored for ≥24 h. Rivaroxaban or dabigatran were administered to prevent thrombus formation, and amiodarone was prescribed for at least 3 months post-procedure. Patients were followed up at 3, 6, 9, and 12 or more months and at any time they felt uncomfortable after the ablation. ECGs, 24-h Holter ECG recordings, and echocardiography were performed at each scheduled follow-up visit. Recurrent AF was considered if symptomatic or asymptomatic AF lasting for more than 30 s after 3 months blanking period was documented on an ECG or Holter study.

Data are reported as means±SD or median and interquartile range for continuous variables and percentages for categorical variables. Continuous variables that had a normal distribution were evaluated using Student's *t*-test, whereas the Mann-Whitney U test was used for non-normally distributed data. Categorical variables and frequencies were compared with the chi-squared test. The baseline clinical variables between patients with or without AF relapse during follow-up were selected for the multivariate analysis. The RIPV parameters including major diameter, minor diameter, and CSA that differed significantly between patients with and without recurrence of AF were tested one at a time by the C statistic. The improvement of discrimination when one RIPV risk marker was added to the risk model was assessed using the C-index and integrated discrimination improvement (IDI) (16), and reclassification was estimated by using continuous net reclassification improvement (NRI). The cumulative proportional probability of AF recurrence for continuous variables was studied using Kaplan-Meier survival analysis with the log-rank test, and the optimal cutoff point was determined using receiver operating characteristic curves. SPSS version 22.0.0 (IBM, USA) and R version 3.4.4 (https://www.r-project.org) were used for statistical analyses, and a two-sided P value of <0.05 was considered statistically significant.

## Results

**Figure 1:**
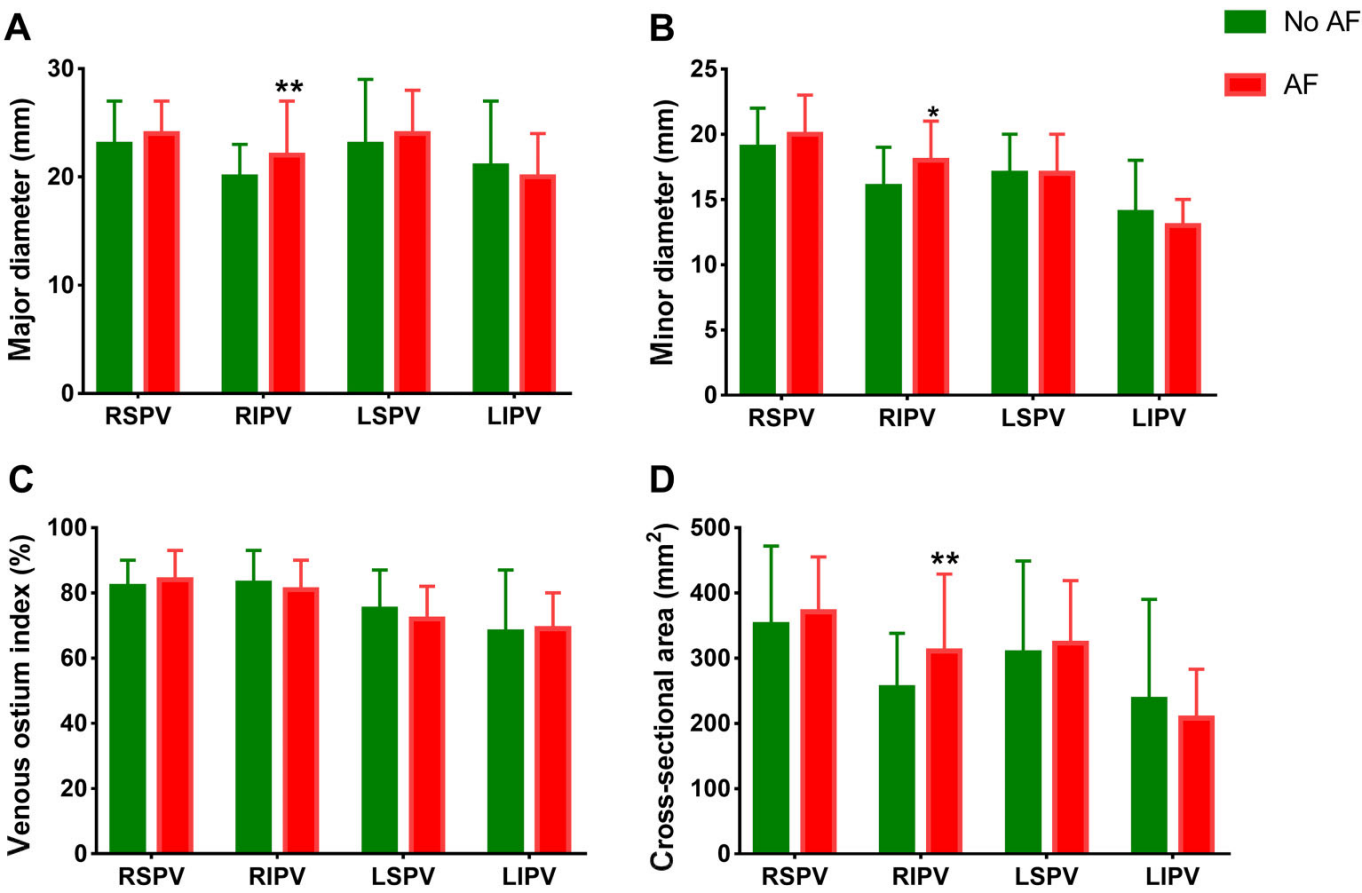
Parameters of pulmonary veins. AF: atrial fibrillation; RSPV: right superior pulmonary vein; RIPV: right inferior pulmonary vein; LSPV: left superior pulmonary vein; LIPV: left inferior pulmonary vein. Data are reported as means±SD *P<0.05, **P<0.01 between groups (Student's *t*-test or Mann-Whitney U test).

**Figure 2:**
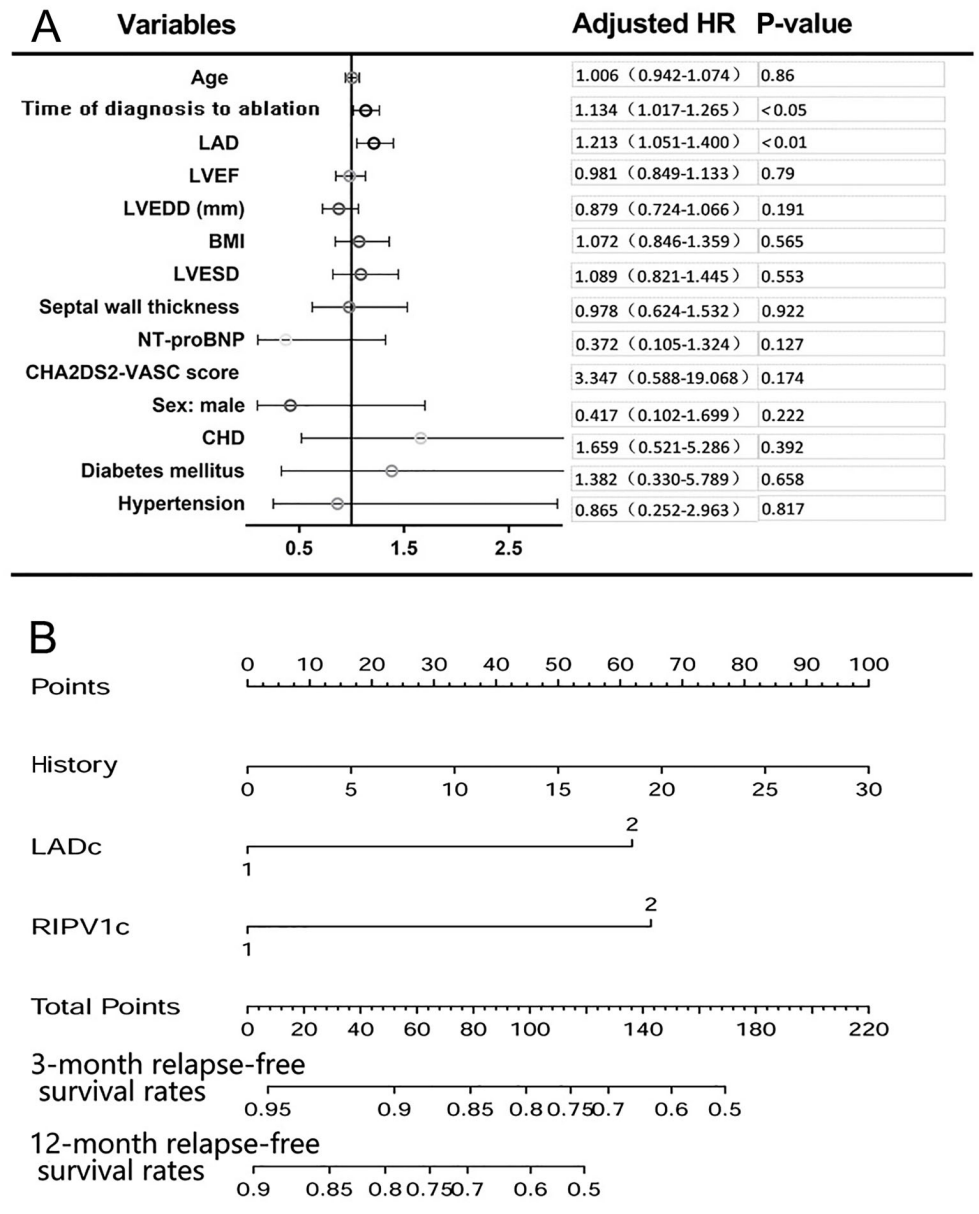
A, Adjusted hazard ratio and 95% confidence intervals for each baseline clinical variable between patients with and without atrial fibrillation recurrence during follow-up. CI: confidence interval; HR: hazard ratio; LAD: left atrial diameter; BMI: body mass index; CHD: coronary heart disease; NT-proBNP: N-terminal pro brain natriuretic peptide; LVEDD: left ventricular end-diastolic diameter; LVESD: left ventricular end-systolic diameter; LVEF: left ventricular ejection fraction. **B**, Instruction for using the nomogram. Draw a line perpendicular from the corresponding axis of each risk factor until it reaches the top line labeled "Points." Sum up the number of points for all risk factors then draw a line descending from the axis labeled "Total Points" until it intercepts each of the survival axes to determine 3- and 12-month relapse-free survival rates. Continuous variables such as LAD and major diameter of right inferior pulmonary vein were converted to categorical variables (LADc and RIPV1c), 0 for no and 1 for yes.

Three patients receiving further radiofrequency ablation for PV conduction recorded from the Achieve catheter were excluded. Thus, ninety-seven patients with paroxysmal AF were included in the study and completed a 12-month follow-up. Ultimately, 28 (28.9%) patients with AF relapse were classified into one group, and the remaining 69 patients without recurrence were included in the other group (Table 1). The RIPV parameters (major diameter, minor diameter, and CSA) and left atrial diameter (LAD) were larger in patients with recurrence than in those without recurrent AF (Table 1 and Figure 1). When baseline clinical variables between the two groups (Table 1) were included in the Cox multivariate clinical hazards model, LAD (HR: 1.213, CI: 1.051–1.400, P<0.01) and the time from first diagnosis of AF to ablation (HR: 1.134, CI: 1.017–1.265, P<0.05) were determined to be independent predictors of AF relapse (Figure 2A).

**Table 1:** Baseline patient characteristics.

Characteristic	No AF (n=69)	AF (n=28)	P
Time from diagnosis to ablation (years)	4.1±4.5	6.1±6.3	0.094
Age (years)	61.2±10.4	64.0±7.6	0.148
Gender (male)	38 (55%)	15 (54%)	0.893
BMI	24.6±2.1	25.0±2.6	0.465
Alcohol use	6 (9%)	3 (11%)	0.715
Smoking	15 (22%)	7 (25%)	0.728
Hypertension	35 (51%)	13 (46%)	0.701
Diabetes mellitus	9 (13%)	5 (18%)	0.537
CHD	21 (30%)	13 (46%)	0.135
CHA_2_DS_2_-VAS_C_ score (≥2)	36 (52%)	20 (71%)	0.091
NT-proBNP (pg/mL)	306 (161–1355)	337 (79–829)	0.238
LAD (mm)	35.1±4.1	37.6±4.2	<0.05
Septal wall thickness (mm)	8.56±1.14	8.56±1.31	0.985
LVEDD (mm)	50.7±6.6	49.9±3.7	0.571
LVESD (mm)	31.1±5.5	31.6±3.5	0.647
LVEF (%)	66±5	65±6	0.426

Data are reported as means±SD, percentage, or median and interquartile range. AF: atrial fibrillation; BMI: body mass index; CHD: coronary heart disease; NT-proBNP: N-terminal pro brain natriuretic peptide; LAD: left atrial diameter; LVEDD: left ventricular end-diastolic diameter; LVESD: left ventricular end-systolic diameter; LVEF: left ventricular ejection fraction. Student's *t*-test, Mann-Whitney U test, or chi-squared test were used to compare the two groups.

The reclassification of patients with *vs* without the occurrence of AF improved significantly when the major LAD (NRI: 0.179, CI: 0.031–0.326, P<0.05) and CSA of RIPV (NRI: 0.122, CI: 0.004–0.240, P<0.05) were added to the model separately. The IDI of patients was significant when the major diameter of RIPV (IDI: 0.071, CI: 0.003–0.138, P<0.05) was included in the AF prediction model. Including the two parameters above (0.766 and 0.758, respectively) in the model increased the C-index moderately (Table 2). Information on the calibration of the models has been provided in Supplementary Figure S1. Based upon these findings, a nomogram was configured (Figure 2B).

**Table 2:** Pulmonary vein parameters for the prediction of improvement in integrated discrimination (IDI) and net reclassification (NRI).

Variable	C-index	IDI (95%CI)	P	NRI (95%CI)	P
Established model	0.691 (0.460–0.923)	Reference		Reference	
+RIPV major D	0.766 (0.534–0.997)	0.071 (0.003–0.138)	<0.05	0.179 (0.031–0.326)	<0.05
+RIPV minor D	0.736 (0.504–0.968)	0.022 (–0.014–0.0585)	0.230	0.050 (–0.024–0.125)	0.185
+RIPV-CSA	0.758 (0.526–0.990)	0.049 (–0.006–0.105)	0.081	0.122 (0.004–0.240)	<0.05

The baseline variables that differed significantly between patients with and without atrial fibrillation recurrence, such as time from diagnosis to ablation and left atrial diameter, were included in the established model. Right inferior pulmonary vein (RIPV) major diameter (D), minor D, and cross-sectional area (CSA) were added to this model as continuous variables. CI: confidence interval.

**Figure 3:**
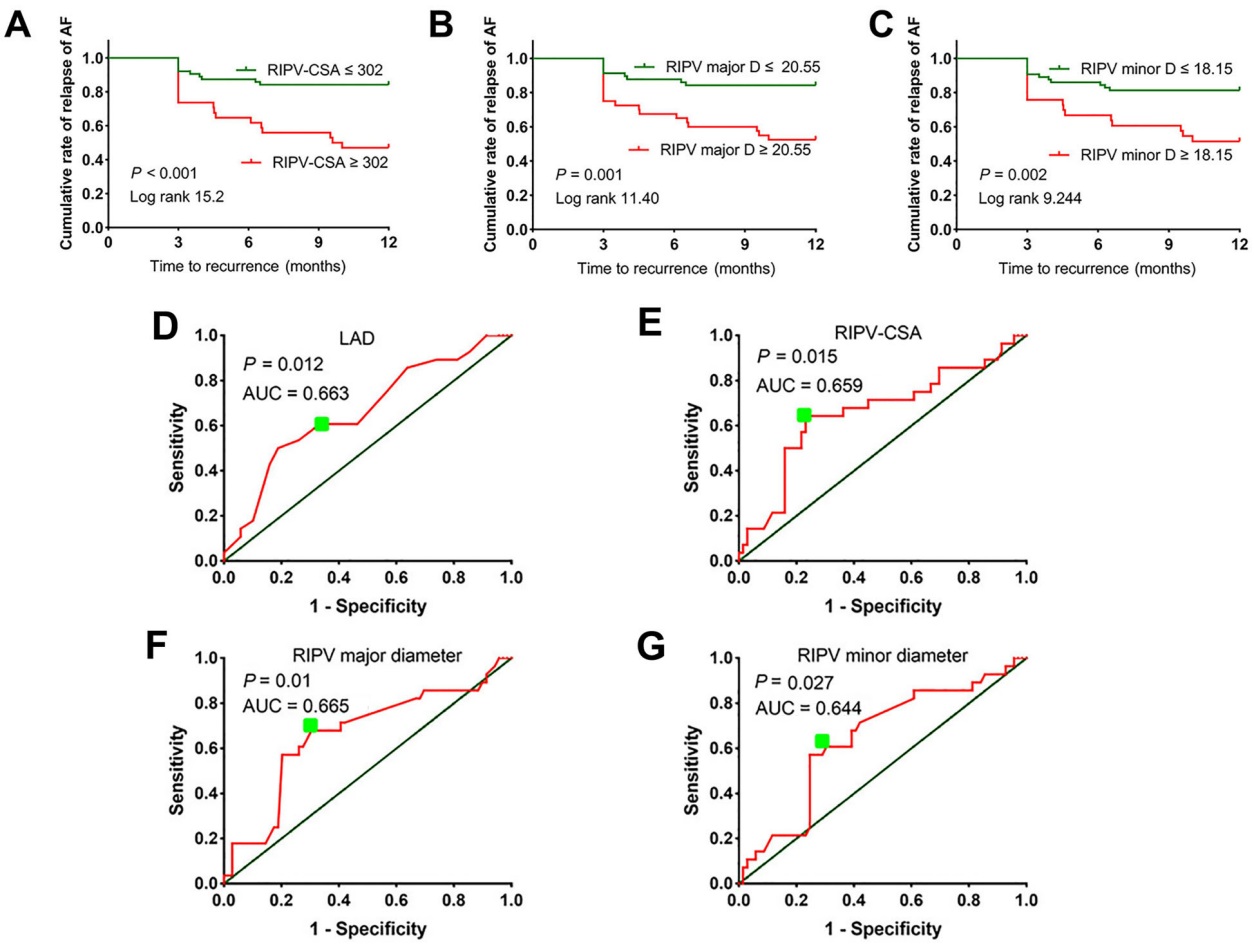
**A–C,** Cumulative proportional probability of atrial fibrillation (AF) recurrence in patients with a right inferior pulmonary vein (RIPV)-cross-sectional area (CSA) ≤ or >302 mm^2^, a RIPV major diameter (D) ≤ or >20.55 mm, and a RIPV minor D ≤ or >18.15 mm. The optimized cutoff points were obtained from the receiver operating characteristic curves. **D–G,** Accuracies of left atrial diameter (LAD), RIPV-CSA, RIPV major diameter, and RIPV minor diameter for predicting AF recurrence are reported as areas under the receiver operating characteristic curves (AUC).

Kaplan-Meier curves were used to illustrate the cumulative proportional probability of AF recurrence (values of major and minor diameters, and CSA of the RIPV less than or equal to the chosen cutoff points *vs* values greater than the chosen cutoff points) in Figure 3A. The accuracies of LAD, major and minor diameters of the RIPV, and CSA of the RIPV for predicting AF relapse are shown as the areas under the receiver operating characteristic curves (Figure 3B).

## Discussion

The main finding of the present study was that among the studied PV parameters, RIPV parameters were the strongest independent predictors of long-term risk of recurrent AF after cryoablation. These parameters, especially the major diameter of the RIPV, predicted AF relapse as well as or even better than LAD, and improved the accuracy of the AF prediction model.

PVs play an essential role in the pathophysiology of AF. Approximately 90% of PVs contain atrial myocardial tissue (17), and the myocardium in PVs is often discontinuous and fibrotic. Abnormalities in the electrical activity in PVs is closely related to AF in many patients (18). Recurrent AF has generally been attributed to the recovery of electrical function after PVI (6,7). Larger PVs with more discontinuous and fibrotic atrial myocardium may have a greater frequency of electrophysiological abnormalities, may be more difficult to isolate, and may be more likely to undergo electrical reconnection than those without these abnormalities.

However, there is no consensus regarding which PV parameters are most strongly associated with AF recurrence in patients undergoing AF ablation. Guler et al. (10) showed that increased RSPV size was a predictor of recurrence. Tsyganov et al. also found that a larger LIPV size was associated with worse long-term outcomes but was not a major risk factor for short- or long-term failure after PVI (9). In the Multicenter Sustained Treatment of Paroxysmal Atrial Fibrillation (STOP-AF) study, there was no correlation between changes in PV parameters and early or late AF recurrence (19). The prognostic value of RIPV parameters were validated in the patients with AF receiving cryoablation in our study. The finding was partially consistent with the research conducted by Shimamoto et al. (20). Their study showed that the value of increased RIPV size for predicting AF recurrence in patients who underwent RF ablation was the most pronounced among PVs ostial area.

Isolation of the RIPV is often thought to be more challenging than isolation of other PV branches mainly due to proximity to the transseptal puncture site and the location of the RIPV itself. Incomplete myocyte damage and local tissue edema may contribute to the appearance of conduction block in patients who may later develop recurrent conduction after myocardial healing. Relapsed AF is highly correlated with lack of scarring in the inferior portion of the RIPV (82%) (21). Studies also confirmed that the majority of new triggers arose from the RIPV, which most commonly reablated during a repeat ablation (22–24).

Increased LAD is widely accepted as a predictor of recurrent AF (17,25–27). Tsao et al. confirmed that the diameters of the PV ostia and LA were increased in patients with AF compared to those without AF (28). Chen et al. (29) have suggested that increased LAD may not be a main risk factor for anatomical remodeling. Research suggested patients with paroxysmal AF had less fibrosis and smaller LA size compared with persistent AF (30). Atrial remodeling of the left atrium in paroxysmal AF was in the early stages, and asymmetrical deformities may occur before chamber dilation (12). Thus, LAD may not have been sensitive enough to reflect an early stage of anatomical remodeling. Potential importance of LA asymmetry index (31), LA sphericity (32), or 3D geometrical models from the segmentation of the LA blood pool (33) were also studied in predicting AF ablation, but clinical series can be difficult. Measurements of LA segments, such as the diameter and CSA of ostium of PV parameters, could provide an easy and generally available way of predicting recurrence.

This study was limited by its relatively small sample size and because it was a single-center study involving a highly selected population. The lack of continuous monitoring might have led to underestimation of the recurrence rate, especially in patients with a short duration of recurrence or asymptomatic recurrent AF. In addition, long-term follow-up after ablation is needed to determine the actual recurrence rate.

## Conclusions

The major diameter and CSA of the RIPV are independent risk factors that can predict AF recurrence at least as accurately as LAD in patients undergoing second-generation cryoballoon ablation.

## Supplementary Material

Click here to view [pdf]
